# Post-hospitalization Short Versus Long Steroid Taper Strategies in Patients With Acute Severe Ulcerative Colitis: A Comparison of Clinical Outcomes

**DOI:** 10.1093/crocol/otae025

**Published:** 2024-04-27

**Authors:** Mohammad Alomari, Pravallika Chadalavada, Sadaf Afraz, Mu’ed AlGhadir-AlKhalaileh, Zoilo K Suarez, Alec Swartz, Mamoon Rashid, Shrouq Khazaaleh, Benjamin L Cohen, Asad Ur Rahman, Mohammad Alomari

**Affiliations:** Department of Gastroenterology and Hepatology, Cleveland Clinic, Cleveland, OH, USA; Department of Gastroenterology and Hepatology, Cleveland Clinic Florida, Weston, FL, USA; Internal Medicine Department, Cleveland Clinic Florida, Weston, FL, USA; Internal Medicine Department, Cleveland Clinic Florida, Weston, FL, USA; Internal Medicine Department, Florida Atlantic University Charles E. Schmidt College of Medicine, Boca Raton, FL, USA; Internal Medicine Department, Cleveland Clinic Florida, Weston, FL, USA; Department of Gastroenterology and Hepatology, Cleveland Clinic Florida, Weston, FL, USA; 5 Internal Medicine Department, Cleveland Clinic Fairview Hospital, Cleveland, OH, USA; Department of Gastroenterology and Hepatology, Cleveland Clinic, Cleveland, OH, USA; Department of Gastroenterology and Hepatology, Cleveland Clinic Florida, Weston, FL, USA

## Abstract

**Background:**

Ulcerative colitis (UC) is a chronic inflammatory colon disease characterized by relapsing flares and remission episodes. However, the optimal steroid tapering strategy in patients hospitalized for acute severe UC (ASUC) remains relatively unknown. We aim to examine the clinical outcomes in patients hospitalized for ASUC regarding variable prednisone taper regimens upon discharge.

**Methods:**

We retrospectively reviewed all adult patients admitted to our facility with ASUC between 2000 and 2022. Patients were divided into 2 groups based on the duration of steroid taper on discharge (< 6 and > 6 weeks). Patients who had colectomy at index admission were excluded from the analysis. The primary outcome was rehospitalization for ASUC within 6 months of index admission. Secondary outcomes included the need for colectomy, worsening endoscopic disease extent and/or severity during the follow-up period (6 months), and a composite outcome as a surrogate of worsening disease (defined as a combination of all products above). Two-sample *t*-tests and Pearson’s chi-square tests were used to compare the means of continuous and categorical variables, respectively. Multivariate logistic regression analysis was performed to identify independent predictors for rehospitalization with ASUC.

**Results:**

A total of 215 patients (short steroid taper = 91 and long steroid taper = 124) were analyzed. A higher number of patients in the long steroid taper group had a longer disease duration since diagnosis and moderate-severe endoscopic disease activity (63.8 vs. 25.6 months, *p* < 0.0001, 46.8% vs. 23.1%, *P* = ≤ .05, respectively). Both groups had similar disease extent, prior biologic therapy, and the need for inpatient rescue therapy. At the 6-month follow-up, rates of rehospitalization with a flare of UC were comparable between the 2 groups (68.3% vs. 68.5%, *P* = .723). On univariate and multivariate logistic regression, escalation of steroid dose within four weeks of discharge (aOR 6.09, 95% CI: 1.82–20.3, *P*  = .003) was noted to be the only independent predictor for rehospitalization with ASUC.

**Conclusions:**

This is the first study comparing clinical outcomes between post-discharge steroid tapering regimens in hospitalized patients for ASUC. Both examined steroid taper regimens upon discharge showed comparable clinical results. Hence, we suggest a short steroid taper as a standard post-hospitalization strategy in patients following ASUC encounters. It is likely to enhance patient tolerability and reduce steroid-related adverse effects without adversely affecting outcomes.

- What is already known? It is well-known that systemic corticosteroid therapy remains the cornerstone of managing ASUC, both in the inpatient setting and in the early post-hospitalization period; despite this, little is known about the optimal steroid tapering strategy, which must balance presumed benefits and potential risks.
- What is new here? This is the first study to compare clinical outcomes between post-discharge steroid tapering techniques for patients hospitalized with ASUC.
- How can this study help patient care? It can guide clinicians to select a strategy that leads to adequate clinical outcomes while avoiding untearable side effects.

## Introduction

Ulcerative colitis (UC) is a common subtype of inflammatory bowel disease (IBD) characterized by contiguous inflammation of the mucosa of the rectum and colon, leading to tenesmus, diarrhea, and gastrointestinal bleeding.^1^ It has a relapsing and remitting fashion and its pathogenesis has been thought to be complex, though dysregulated immune response, intestinal microbiota alterations, together with multiple environmental and genetic factors have been hypothesized to be related to its development.^2,3^ In recent years, an increase in incidence and prevalence have been reported^4,5^ with comorbid significant societal, financial, and direct healthcare burden,^6–10^ the last of which is thought to be driven by disease-related hospitalizations.^11^

Acute severe UC (ASUC) was defined by Truelove and Witts as 6 or more bloody bowel movements per day in the presence of signs of systemic toxicity, including tachycardia, pyrexia, anemia, and/or elevated inflammatory markers.^12^ ASUC is considered a medical emergency with significant health risks and increased short- and long-term colectomy rates.^1,13^ It has been estimated to occur in nearly a quarter of patients with UC, typically requiring inpatient management with intravenous glucocorticoids and if needed rescue therapies such as infliximab or intravenous cyclosporine to dampen the immune response, and reduce the likelihood of requiring colectomy.^14^

In the early 1900s, the mortality rate associated with ASUC was as high as 75%, decreasing substantially after the introduction of corticosteroids and early-colectomy for steroid-refractory cases to < 1% in tertiary care centers.^15^ Intravenous steroids (Methylprednisolone 60 mg per day or hydrocortisone 100 mg three to four times a day) are effective in achieving remission in 65%–69% of cases^16^ and improving in-hospital mortality.^17^ Concerns about their safety profile have made them less attractive and hence led to the development of formulations with lower systemic bioavailability, as well as steroid-sparing therapeutic techniques.^17^ It is generally recommended that corticosteroids be tapered over several weeks, first to prevent a rapid relapse, second as a bridging agent during the introduction or optimization of other therapies, and third to allow the hypothalamic–pituitary–adrenal axis to resume its normal function.^18^ Early relapse has been associated with short courses (ie, 3 weeks) and modest initial doses (ie, 15 mg prednisolone) of oral corticosteroids.^19^ In addition, extended periods of corticosteroids are ineffective in maintaining remission^20–22^ leading to a longer disease course and exposure to side effects.^23,24^

Management of patients hospitalized for ASUC is challenging,^25^ and the response to steroids is assessed daily during the first 3 to 5 days.^26,27^ The optimal steroid tapering strategy post-discharge remains unknown. A starting dose of prednisolone 40 mg per day is as effective as higher doses.^28^ However, the appropriate duration of tapering is unknown, as short courses of oral corticosteroids are associated with early relapse, while longer tapering is not associated with better clinical results. In addition, this may predispose patients to systemic side effects, such as opportunistic infections, psychiatric disturbances, osteoporosis, myopathy, and glucose intolerance, among others.^29^

Despite the well-defined efficacy and role of steroids in ASUC, there is division amongst international organizations concerning the optimal tapering strategy. The British Society of Gastroenterology guidelines recommend that patients who have appropriately responded to intravenous corticosteroids can have them tapered over 6 to 8 weeks, starting at 40 mg of prednisolone followed by the introduction of maintenance therapy.^30^ Conversely, the American College of Gastroenterology guidelines state that the optimal tapering regimen has not been established but suggests that the dose of corticosteroids is usually reduced over 8 to 12 weeks.^31,32^

In this study, we aim to compare clinical outcomes between post-discharge steroid tapering strategies for patients admitted to ASUC.

## Methods

Subjects were eligible in the study if all of the following inclusion criteria were met: (1) male or female patients age ≥ 18 years, (2) history of UC previously on clinical remission, and (3) admission for ASUC, as defined by the Truelove and Witts criteria, between the year 2000 and 2022. Patients who had colectomy at index admission were excluded from the analysis. We retrospectively reviewed all the discharge summaries from electronic medical records of adult patients admitted to Cleveland Clinic with ASUC between 2000 and 2022. Patients were divided into 2 groups based on the duration of steroid taper on discharge (≤ 6 and ≥ 6 weeks). The study protocol was approved by the institutional review board of the Cleveland Clinic Foundation.

### Study Variables and Assessments

We retrospectively collected the following data on all our included patients: Age, sex, race, smoking status, and duration of UC. Baseline disease course was assessed by collecting the following data before index admission; steroid use in the past 6 months, prior biologic therapy, number of biologics used in the past, and extent and severity of endoscopic disease. Other variables of interest included length of stay, C-reactive protein (CRP), and fecal calprotectin at 1 week and at 6 months after index hospitalization, time to new biologic initiation, steroid dose escalation within 4 weeks of discharge, and reason for readmission. Prednisone was the only form of steroid used in both groups.

### Outcomes

The primary outcome was rehospitalization for ASUC within 6 months of index admission. Secondary outcomes of interest were the need for colectomy, worsening endoscopic disease extent and/or severity during the follow-up period (6 months), and a composite outcome as a surrogate of worsening disease (defined as a combination of all products above).

### Statistical Analysis

Continuous variables were presented as mean (± standard deviation), and categorical variables as counts and frequency. Univariable analysis was performed to assess differences between study groups. A 2-sample *t*-test was used to compare medians of continuous variables. Pearson’s chi-square tests were used to compare categorical variables. Multivariate logistic regression analysis was performed to identify independent predictors for rehospitalization with ASUC. All statistical analyses were performed using the STATA, version 17.0 (StataCorp, USA). A *P* -value < .05 was considered statistically significant.

## Results

### Baseline Characteristics

A total of 215 patients (short steroid taper = 91 and long steroid taper = 124) were analyzed. Patient and disease characteristics are listed in Table 1. There were no significant differences in age, sex distribution, and race between the 2 study groups. A higher number of patients in the long steroid taper group had a longer disease duration since diagnosis and moderate-severe endoscopic disease activity (63.8 vs. 25.6 months, *P* < .0001, 46.8% vs. 23.1%, *P* = ≤ .05, respectively). Both groups had similar disease extent and prior biologic therapy. 37.4% of the patients in the short taper group and 41.1% in the long taper group were on biologic therapy prior to index hospitalization (*P* = .206).

**Table 1.** Baseline demographics of ulcerative colitis patients treated with short and long steroid taper regimens.

Factor	Short steroid taper (*n* = 91)	Long steroid taper (*n* = 124)	*P* value
Age (mean—years.)	43.7 ± 18.6	39.9 ± 15.9	.108
Age at UC diagnosis (mean—years.)	32.5 ± 17.3	31.0 ± 15.7	.509
Male (*n*%)	33 (36.3%)	54 (43.5%)	.282
Race (*n*%)			
Caucasian	55 (60.4%)	70 (56.5%)	.083
African American	5 (5.5%)	13 (10.5%)	.237
Hispanic	2 (2.2%)	8 (6.5%)	.169
Smoking (*n*%)			.066
Never	33 (36.3%)	34 (27.4%)	
Current	54 (59.3%)	89 (71.8%)	
Past	4 (4.4%)	1 (0.8%)	
Disease duration (months)	25.6 ± 9.59	63.8 ± 22.9	.0000
LOS (days)	4.34 ± 3.59	5.35 ± 4.73	.090
Steroid use in the past six months	53 (58.2%)	76 (61.3%)	.652
The extent of disease at index hospitalization (n)	32	61	.159
Proctosigmoiditis	6 (6.6%)	12 (9.7%)	
Left-sided colitis	9 (9.9%)	23 (18.5%)	
Colitis proximal to the splenic flexure	13 (14.35%)	25 (20.2%)	
Prior biologic therapy (*n*%)	35 (38.5%)	59 (47.6%)	.206
Prior anti-TNF therapy (*n*%)	34 (37.4%)	51 (41.1%)	.160
Type of biologic agent			
Infliximab	27 (29.7%)	36 (29.0%)	.162
Adalimumab	15 (16.5%)	22 (17.7%)	.645
Vedolizumab	11 (12.1%)	25 (20.2%)	.275
Ustekinumab	1 (1.1%)	4 (3.2%)	.404
Golimumab	1 (1.1%)	2 (1.6%)	.876
Certolizumab	0 (0.0%)	1 (0.8%)	.435
No. of biologics used in the past (*n*)			
One agent	20 (21.9%)	33 (26.6%)	.971
≥ 2 agents	15 (17.6%)	26 (21.0%)	.220
Endoscopic disease activity at Index hospitalization (*n*)	31	61	.004
Mayo 2	11 (12.1%)	24 (19.4%)	
Mayo 3	10 (11.0%)	34 (27.4%)	
CRP within 1 week of discharge (mean—mg/dl.)	10.8 ± 2.5	11.5 ± 3.8	.868
Fecal calprotectin within one week of discharge (mean—ug/g)	2993.3 ± 2075.3	2721.2 ± 1748.3	.6647
Inpatient rescue therapy (%)	11 (12.1%)	21 (16.9%)	.324
In-hospital colectomy (%)	0 (0.0%)	0 (0.0%)	—

### Disease Characteristics and Hospital Course at Index Hospitalization

A total of 14.4% of the patients in the short steroid group and 20.1% in the long steroid taper group had colitis proximal to the splenic flexure. There was no statistical difference in the endoscopic disease extent between the 2 groups. A significantly higher proportion of patients who were started on long steroid taper had severe endoscopic disease activity (mayo endoscopic score of 3; 27.4% vs. 11.0%, *P* = .004). The mean length of stay was among the 2 study groups (4.34 vs. 5.35 days, *P* = .090) respectively. The need for inpatient rescue therapy was comparable among the 2 study groups (12.1% vs. 16.9%, *P* = .324).

### Outcomes

At the 6-month follow-up, rates of rehospitalization with a flare of UC were comparable between the 2 groups (65.1% vs. 68.5%, *P* = .723). There was no significant difference in the change in endoscopic disease extent and severity among the 2 groups during the study follow-up period. (Table 2). A higher number of patients in the long steroid taper group followed up in the office within 4 weeks of discharge (77.4% vs. 64.8%, *P* = .042). There was no significant difference in the time to new biologic initiation (130 vs. 91.3 days, *P* = .639) and the proportion of patients who started biologic therapy beyond 6 weeks following discharge. 27.4% of patients who received short steroid taper received dose escalation within 4 weeks of discharge, as opposed to only 12.2% among patients treated with long steroid taper (*P* = .004).

**Table 2.** Outcomes in ulcerative colitis patients treated with short and long steroid taper regimens.

Factor	Short steroid taper (*n* = 91)	Long steroid taper (*n* = 124)	*P* value
Follow-up within 4 weeks of discharge (%)	59(64.8%)	96 (77.4%)	.042
Time to new biologic initiation (days)	130.8 ± 40.5.	91.3 ± 43.7	.639
Biologic initiation beyond 6 weeks (%)	22(24.1%)	50 (40.3%)	.126
CRP within 6 months from index admission	4.28 ± 6.73	2.95 ± 4.01	.145
FC within 6 months from index admission	2029.9 ± 1530.7	1609.2 ± 1594.1	.499
Steroid dose escalation within 4 weeks of discharge (%)	25 (27.4%)	15 (12.1%)	.004
Readmission within 6 months (%)	43 (47.2%)	54 (43.5%)	.826
Reason for readmission (n)	43	54	
The flare of UC	28 (65.1%)	37 (68.5%)	.723
Sepsis	4 (9.3%)	3 (5.5%)	.479
Surgery	2 (4.6%)	6 (11.1%)	.251
C. Diff colitis	1 (2.3%)	1 (1.9%)	.187
Others	8 (18.6%)	7 (12.9%)	.445
Endoscopic disease assessment			
Change in mayo score from baseline (*n*)	23	48	
Worsening	3 (13.1%)	3 (6.3%)	.366
Improved	8 (34.7%)	24 (50.0%)	.180
Stable	13 (56.5%)	21 (43.8%)	.404
Change in disease extent from baseline (*n*)	19	42	
Worsening	3 (15.7%)	3 (7.1%)	.366
Improved	9 (47.3%)	14 (33.3%)	.514
Stable	7 (36.8%)	25 (59.5%)	.059
Composite outcome (%)	36 (39.6%)	52 (41.9%)	.726

### Predictors of Rehospitalization With ASUC

We performed multivariate logistic regression with an adjusted odds ratio (aOR) to identify predictors of rehospitalization with ASUC. On univariate and multivariate logistic regression, escalation of steroid dose within 4 weeks of discharge (aOR 6.09, 95% CI: 1.82–20.3, *P*  = .003) was noted to be the only independent predictor for rehospitalization with ASUC. The duration of steroid taper was not associated with a readmission for UC flare (aOR 1.16, 95% CI: 0.49–2.72, *P* = .723). The results of univariate and multivariate analysis with aOR are shown in Tables 3 and 4, respectively.

**Table 3.** Univariate analysis of predictors of readmission for an ulcerative colitis flare.

Factor	Odds ratio	95% Conf. Interval	*P* value
Long steroid taper	1.16	0.49, 2.72	.723
Disease duration	1.00	0.98, 1.02	.614
The initial dose of Prednisone	1.02	0.95, 1.11	.472
Initial prednisone dose > 40 mg	2.13	0.40, 11.2	.370
Clinic Follow-up within 4 weeks	0.67	0.25, 1.83	.443
Steroid use in the last 6 months	0.63	0.26, 1.56	.328
Steroid dose escalation within 4 weeks of discharge	5.29	1.66, 16.8	.001
Inpatient rescue therapy	1.96	0.50, 7.62	.327

**Table 4.** Multivariate analysis of predictors of readmission for an ulcerative colitis flare.

Factor	Odds ratio	95% Conf. Interval	*P* value
Steroid dose escalation within 4 weeks of discharge	6.09	1.82, 20.3	.003

Data not publicly available.

## Discussion

This is, to the best of our knowledge, the first study to compare clinical outcomes between post-discharge steroid tapering strategies for ASUC hospitalized patients. Short and extended steroid taper regimens yielded comparable clinical outcomes upon discharge. At the 6-month follow-up, rehospitalization rates for UC flare-ups were comparable between the 2 groups (68.3% vs. 68.5%, *P* = .723). Also, the change in severity and endoscopic disease extent, need for colectomy during the follow-up period and the composite outcome showed no significant difference between the 2 groups (39.6% vs 41.9%; *P* = .726).

The efficacy of corticosteroids for inducing remission in moderate-to-severely active UC is undisputed; however, to minimize their side effects, they should be administered at the lowest effective dose for the shortest duration possible, with early consideration of corticosteroid-sparing agents. A positive clinical and endoscopic response is expected after 2 weeks,^33,34^ and patients who have not reacted by that time are considered corticosteroid-resistant for which therapeutic alternatives should be sought.^35^ In our study, a univariate and multivariate logistic regression analysis revealed that an increase in the dose of steroids within 4 weeks of discharge was the only independent predictor of rehospitalization in patients with ASUC (OR 6.09, 95% CI: 1.73–20.3, *P* = .003). This is particularly important in interpreting our findings, as patients who required steroid dose escalation within 4 weeks of discharge were more prevalent in the short taper group (27.4% vs. 12.2%, *P* = .004). Despite this, and acknowledging that the long steroid taper group likely had more severe disease at baseline (considering disease duration, extent, use of prior biological therapy, Mayo endoscopic score, and need for rescue therapy), there was no statistically significant difference in the rehospitalization rate. A potential explanation for this paradox, is a higher proportion of steroid-resistant patients in the short steroid taper group as dosage escalation happened within the first 4 weeks of therapy, although this is hard to confirm with the current study design. Therefore, early clinical evaluation should be performed to identify patients who are corticosteroid non-responsive, to pursue surgical or rescue therapy in a timely manner.

Steroid stewardship is necessary to avoid unnecessary side effects,^23^ as well as to consider switching to non-steroidal agents.^24^ Hyperglycemia is one of the most common metabolic derangements associated with corticosteroids, and the incidence of diabetes mellitus is proportional to its dose and duration.^36^ Prolonged corticosteroid courses have also been linked with hypercholesterolemia, hypertriglyceridemia, and hepatic steatosis.^37,38^ Mood disorders have been reported in up to 60% of patients, with serious psychotic and affective symptoms occurring in around 5%.^39^ Similarly, IBD patients on corticosteroids are at an increased risk of common, opportunistic, and postoperative infections.^40–42^ Data from 71 controlled trials evaluating corticosteroid therapy for different indications revealed that patients taking more than 40 mg of prednisone per day were at the greatest risk for infection, with no increased risk at doses of less than 10 mg per day.^43^ Additionally, decreased bone mineral density, including osteopenia and osteoporosis has been reported in a significant number of patients with IBD.^44,45^ Finally, even though our findings showed no difference in terms of adverse effects between the 2 groups, the existing literature is enough to suggest that shorter steroid courses should be preferred whenever possible to avoid them.

Our study had certain limitations. Firstly, our sample size was small, and the selection of patients was not random; individual circumstances may have favored either approach at the time. Additionally, our analysis did not include the index hospitalization albumin level, which might have varied between groups. Also, the clinical picture of the group receiving a longer taper was less favorable as detailed above. The extended duration of this study allows comprehensive evaluation of disease activity; however, it is important to interpret the results considering the advances in UC pharmacotherapy during the particular period studied.

In conclusion, to confirm our findings a double-blind clinical study with a larger sample would be recommended. It would also be worthwhile to examine infectious complications as a secondary outcome and the timing of the maintenance therapy initiation post-achievement of remission. Meanwhile, with careful post-discharge monitoring and as-needed dose escalation, shorter (ie, 6-week) steroid taper strategies are suggested.

## Supplementary Material

otae025_suppl_Supplementary_Materials
